# Successful management of chronic eosinophilic pneumonia triggered by immune checkpoint inhibitor: a case report and literature review

**DOI:** 10.3389/fimmu.2025.1615531

**Published:** 2025-07-22

**Authors:** Yasuhito Suzuki, Junpei Saito, Satoshi Kubota, Masakazu Ikeda, Mami Rikimaru, Ryuki Yamada, Takahiro Kumanaka, Ryutaro Tanaka, Kentaro Kazama, Koshi Saito, Rina Harigane, Riko Sato, Hikaru Tomita, Natsumi Watanabe, Takashi Umeda, Ryuichi Togawa, Yuki Sato, Takefumi Nikaido, Xintao Wang, Kenya Kanazawa, Yoshinori Tanino, Shigeyuki Murono, Yoko Shibata

**Affiliations:** ^1^ Department of Pulmonary Medicine, Fukushima Medical University School of Medicine, Fukushima, Japan; ^2^ Department of Otolaryngology-Head and Neck Surgery, Fukushima Medical University School of Medicine, Fukushima, Japan

**Keywords:** eosinophilic pneumonia, fractional exhaled nitric oxide, immune checkpoint inhibitors, immune-related adverse events, blood eosinophil, corticosteroid, interstitial lung disease

## Abstract

Immune checkpoint inhibitors (ICIs) enhance antitumor immunity by blocking inhibitory immune signals, but can lead to immune-related adverse events (irAEs). Therefore, effective management of irAEs is crucial during ICI therapy. We report the case of a 50-year-old man who was referred to our department due to cough and abnormal chest shadows. He was diagnosed with hypopharyngeal cancer, and underwent chemoradiotherapy, resulting in complete remission. However, metastatic tumors were detected, and partial lung resection was performed. After one-year, new metastatic tumors and pleural dissemination were identified. Therefore, treatment with pembrolizumab was initiated. After the treatment with pembrolizumab, chest imaging revealed ground-glass opacity (GGO). Laboratory tests showed elevated eosinophils, and fractional exhaled nitric oxide (FeNO). The findings of bronchoscopy revealed eosinophilic infiltration and intraluminal fibrosis, consistent with chronic eosinophilic pneumonia (EP). Based on these findings, he was diagnosed with pembrolizumab-induced chronic EP. Pembrolizumab was temporarily discontinued, and oral corticosteroids (OCS) were initiated. After the treatment of OCS, his symptoms and GGO were dramatically improved. Subsequently, pembrolizumab was resumed, and the hypopharyngeal cancer remains stable without recurrence of EP. This report presents the first pembrolizumab-induced chronic EP during treatment for hypopharyngeal cancer. The chronic EP was effectively managed with systemic corticosteroid therapy. Furthermore, pembrolizumab was resumed with close monitoring of blood eosinophil counts and FeNO levels, without worsening of EP. The results of the current case suggest that ICI-induced chronic EP is manageable, and in cases where ICI therapy exhibits significant efficacy against cancer, its treatment may be continued with careful monitoring of these parameters.

## Introduction

Immune checkpoint inhibitors (ICIs) have revolutionized cancer treatment, and are now widely used for various cancers. Anti-programmed cell death 1 (PD-1), anti-programmed cell death ligand 1 (PD-L1), and cytotoxic T-lymphocyte-associated protein 4 (CTLA-4) are the most frequently used ICIs (1). The mechanism of ICIs involves blocking inhibitory signals that suppress immune responses, thereby enhancing T cell activity to eliminate tumor cells (2). However, their use has revealed a variety of immune-related adverse events (irAEs), most of which are manageable, but some can be unexpected or severe (3). ICIs may disrupt self-tolerance, leading to the emergence of immune-mediated responses against nontumor cells (4). These irAEs can affect any organ system and are classified as part of a broad spectrum of autoimmune disorders induced by monoclonal antibodies targeting immune regulators (5). Among them, ICI-related interstitial lung disease (ILD) is clinically significant, encompassing a broad spectrum of irAEs, ranging from mild to severe, progressive, and potentially life-threatening conditions (1). Therefore, accurate diagnosis and proper management strategies for these events, based on effective information sharing, are crucial, since they may significantly impact the safety, subsequent treatment options, and overall prognosis of cancer patients. Eosinophilic pneumonia (EP) comprises a heterogeneous group of ILD characterized by prominent infiltration of eosinophils into the pulmonary interstitium and alveoli (6). EP is classified into acute EP and chronic EP (6), and can have various causes, including medications or other toxins (7).

Herein, we report, to the best of our knowledge, the first case of chronic EP triggered by pembrolizumab, a PD-1 inhibitor, in a patient with hypopharyngeal cancer, along with a review of the relevant literature.

## Case presentation

**Figure 1:**
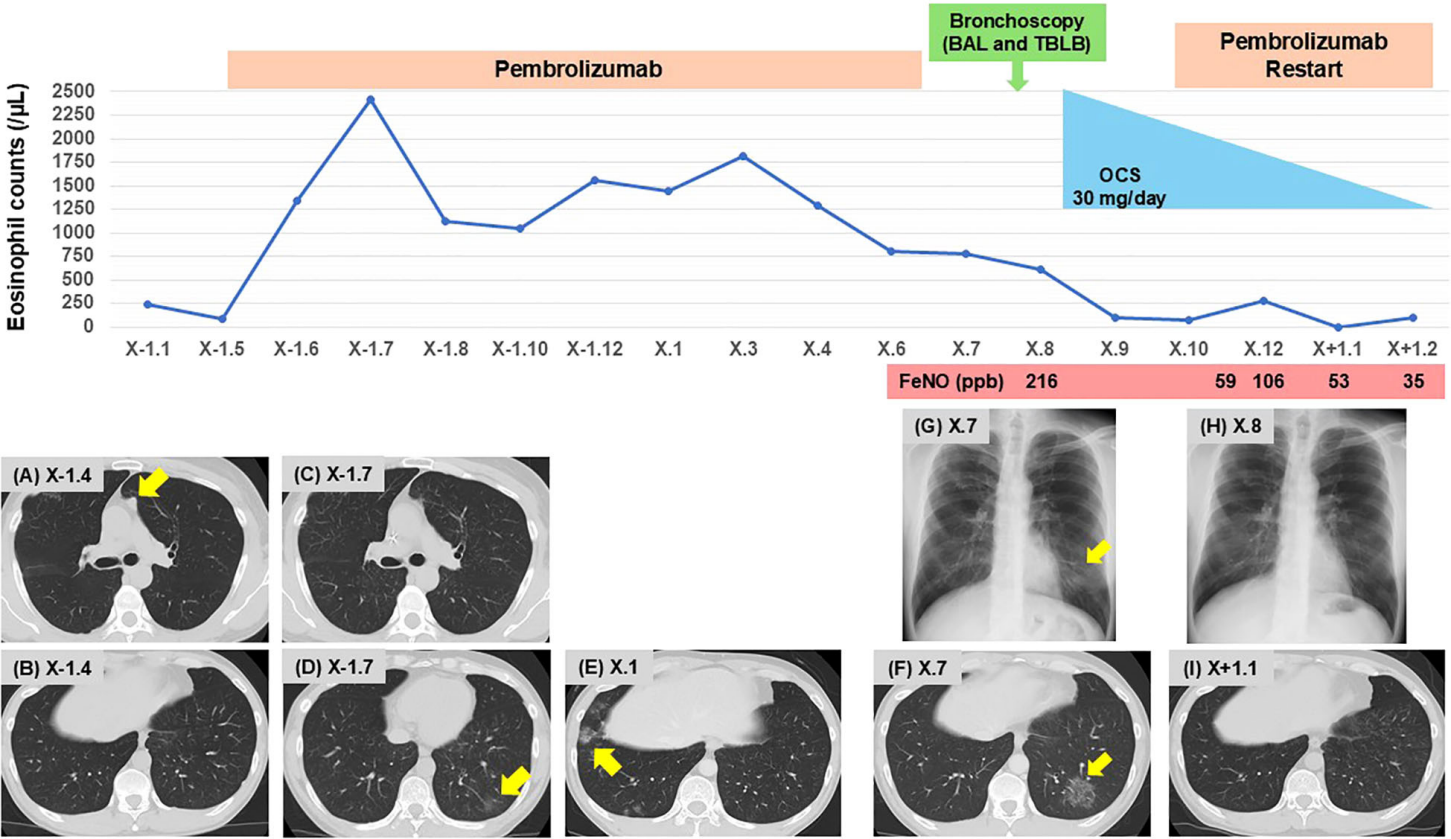
Clinical course of the patient. Changes in eosinophil counts, and imaging findings on chest CT **(A–F, I)** and radiography **(G, H)**. **(A, B)** Pre-treatment with pembrolizumab; **(A)** metastatic lung nodules in the left upper lobe (S^1 + 2^, arrow), and **(B)** no signs of pneumonia. **(C, D)** Two months after initiating pembrolizumab; **(C)** improvement of metastatic lung nodules, and **(D)** onset of GGO in the left lower lobe (arrow). **(E)** Eight months after initiating pembrolizumab; onset of GGO in the right lower lobe (arrow). **(F)** Fourteen months after initiating pembrolizumab; onset of GGO in the left lower lobe (arrow). **(G)** Upon presentation to our clinic; GGO in the left lower lung field (arrow). **(H, I)** Following OCS therapy; improvement of GGO in the left lower lobe. BAL, bronchoalveolar lavage; CT, computed tomography; GGO, ground-glass opacity; FeNO. fractional exhaled nitric oxide; OCS, oral corticosteroids; TBLB, transbronchial lung biopsy.

A 50-year-old man was referred to our department due to cough and abnormal chest shadows. He had a 45 pack-year smoking history, and no history of respiratory or allergic disorders. At the age of 46, he was diagnosed with hypopharyngeal cancer (cT4aN3bM0, cStage IVB), based on the Japanese Clinical Practice Guidelines of Head and Neck Cancer, 4th edition (8). He underwent chemoradiotherapy consisting of three courses of cisplatin (100 mg/m² every 3 weeks) and a total radiation dose of 70 Gy, resulting in complete remission. At the age of 48, metastatic tumors were detected in the posterior segment of the right upper lobe (S^2^ segment), and partial lung resection was performed under thoracoscopy. At the age of 49, new metastatic tumors in the left upper lobe (S^1 + 2^ segment) and left pleural dissemination were identified. Therefore, treatment with pembrolizumab at a dose of 200 mg every 3 weeks was initiated as the first-line therapy for recurrent and metastatic hypopharyngeal cancer based on a combined positive score of 5 for PD-L1 in the tumor specimen obtained from the resected lung (9). Pre-treatment chest computed tomography (CT) showed no significant findings other than left metastatic lung nodules and pleural dissemination (**Figures 1A, B**). One month after starting pembrolizumab therapy, peripheral blood eosinophil levels began to gradually increase without any symptoms (**Figure 1**). A CT scan taken 2 months after treatment initiation showed a dramatic improvement in the metastatic tumors and left pleural dissemination (**Figure 1C**), while also revealing a ground-glass opacity (GGO) in the left lower lobe (**Figure 1D**). As the patient remained asymptomatic, the GGO was observed without any therapeutic intervention. Notably, while the GGO had disappeared on the CT scan obtained 8 months after treatment initiation, a new GGO had appeared in the right lower lobe (**Figure 1E**), and monitoring was continued without treatment. Of note, after 14 months of pembrolizumab therapy, corresponding to 18 courses, the infiltrative shadow in the right lower lobe had spontaneously disappeared, whereas a new GGO had appeared in the left lower lobe (**Figure 1F**), which was accompanied by coughing. The patient was subsequently referred to our outpatient clinic.

Chest radiograph on initial presentation showed a GGO in the left lower lung field (**Figure 1G**). Physical examination revealed no crackles or wheezing, and the oxygen saturation was 98% on room air. As shown in **Table 1**, laboratory examinations revealed increased blood eosinophils of 774/µL (maximum of 2,418/µL during pembrolizumab therapy), immunoglobulin E of 244 IU/mL, and surfactant protein D (SP-D) of 128 ng/mL (normal range < 110 ng/mL). C-reactive protein was 0.24 mg/dL, and sialylated carbohydrate antigen KL-6 was 354 U/mL (normal range < 500 U/mL), which were both within the normal range. There was no elevation in antinuclear antibodies, proteinase 3 anti-neutrophil cytoplasmic antibodies, or myeloperoxidase anti-neutrophil cytoplasmic antibodies. Pulmonary function tests were within the normal range, including small airway parameters such as V50/V25, whereas fractional exhaled nitric oxide (FeNO) level was remarkably elevated at 216 ppb.

**Table 1:** Laboratory data obtained at the time of the outpatient clinic visit.

Hematology	Values	Units	Normal range	Units	Blood chemistry	Values	Units	Normal range	Units
White blood cell	8600	/μL	3300–8600	/μL	Total protein	6.6	g/dL	6.6–8.1	g/dL
Neutrophils	76	%	44–74	%	Albumin	3.8	g/dL	4.1–5.1	g/dL
Lymphocytes	12	%	20–50	%	AST	16	U/L	13–30	U/L
Monocytes	3	%	1–14	%	ALT	13	U/L	10–42	U/L
Eosinophil	9	%	0–6	%	LDH	241	U/L	124–222	U/L
Red blood cell	544	×10^4^/μL	435–555	×10^4^/μL	Total bilirubin	0.5	mg/dL	0.4–1.5	mg/dL
Hemoglobin	15.2	g/dL	13.7–16.8	g/dL	BUN	14	mg/dL	8–20	mg/dL
Hematocrit	46.6	%	40.7–50.1	%	Creatinine	1.06	mg/dL	0.65–1.07	mg/dL
Platelet	26.3	×10^4^/μL	15.8–34.8	×10^4^/μL	Na	138	mEq/L	138–145	mEq/L

Immunologic test					K	4.8	mEq/L	3.6–4.8	mEq/L
IgG	878	mg/dL	861–1747	mg/dL	Cl	103	mEq/L	101–108	mEq/L
IgA	81	mg/dL	93–393	mg/dL	CRP	0.24	mg/dL	< 0.30	mg/dL
IgM	43	mg/dL	33–183	mg/dL	BNP	6.4	pg/mL	< 18.4	pg/mL
IgE	244	IU/mL	< 169	IU/mL	Glucose	97	mg/dL	73–109	mg/dL
					HbA1c (NGSP)	6.1	%	4.9–6.0	%
					KL-6	354	U/mL	< 500	U/mL
					SP-D	128	ng/mL	< 110	ng/mL
					PR-3 ANCA	<0.6	U/mL	< 2.0	U/mL
					MPO-ANCA	0.3	U/mL	< 3.5	U/mL

Spirometry and FeNO	Values	Units	Normal range	Units
FVC	4.77	L	N/A	L
%FVC	119	%	> 80	%
FEV_1_	3.78	L	N/A	L
%FEV_1_	111	%	> 80	%
FEV_1_/FVC	79.3	%	> 70	%
FeNO	216	ppb	< 22	ppb

BALF findings (left B^9^)	Values	Units	Normal range	Units
Recovery (rate)	45/150 (30)	mL (%)	N/A	mL (%)
Total cell count	25.1	x 10^4/^mlBALF	N/A	x 10^4/^mlBALF
Macrophages	22.1	%	78–98	%
Lymphocytes	17.9	%	1–20	%
Neutrophils	29.7	%	0–0.5	%
Eosinophils	30.3	%	0–0.6	%
CD4/CD8 ratio	0.4		0.4–1.9	
Bacterial culture	Negative		N/A	
Acid-fast bacillus culture	Negative		N/A	

**Figure 2:**
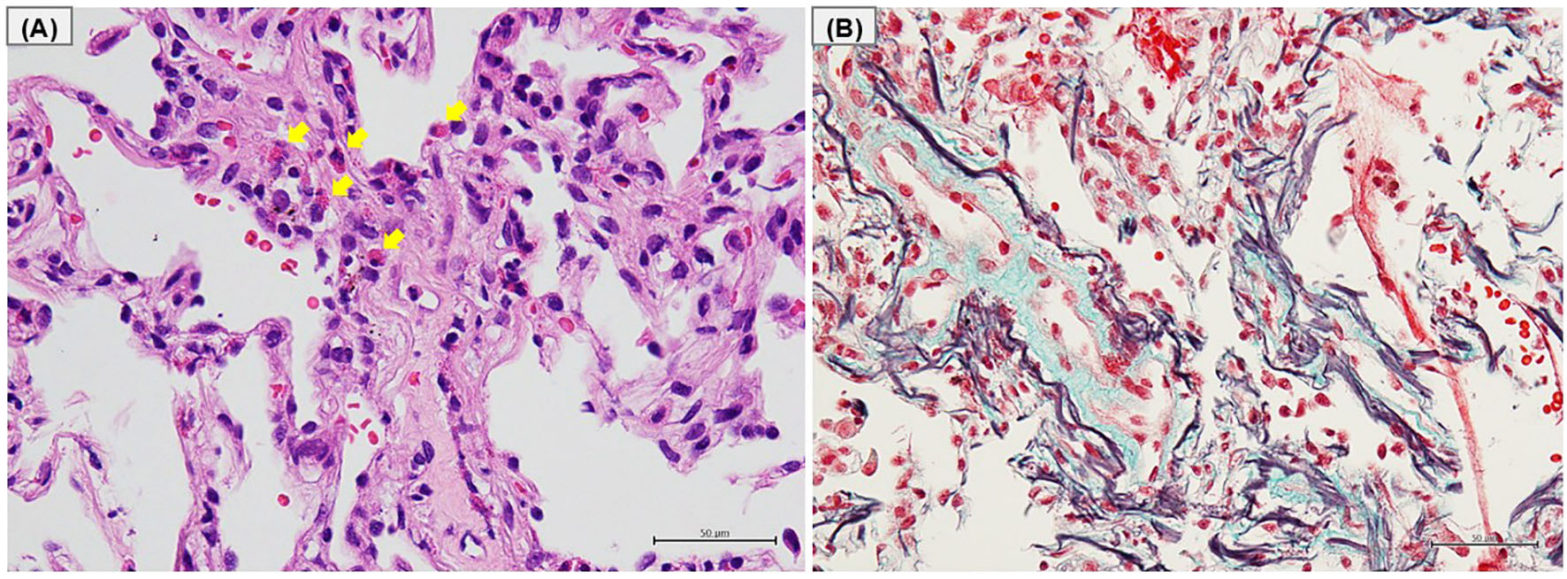
Histological findings of transbronchial lung biopsy. **(A)** Hematoxylin-eosin staining and **(B)** Elastica-Masson staining of the left lower lobe tissue obtained by transbronchial lung biopsies. **(A)** Eosinophilic infiltration with degranulation in the blood vessels and around the alveoli (arrows). **(B)** Deposition of collagen fibers on the luminal side of the alveoli.

Bronchoscopy was performed, and bronchoalveolar lavage fluid (BALF) from the left lower lobe revealed an increased total cell count (25.1 x 10^4^/mlBALF) and a high proportion of eosinophils (30.3%), with no malignant cells or microorganisms detected. Transbronchial lung biopsy samples obtained from left B^9^ and B^10^ showed eosinophilic infiltration with degranulation, not only within the blood vessels but also around the alveoli (**Figure 2A**). In addition, Elastica-Masson staining revealed intraluminal fibrosis (**Figure 2B**). These findings were all indicative of chronic EP rather than acute EP. Based on the clinical, laboratory, radiological, and pathological findings, along with pembrolizumab treatment, the patient was finally diagnosed with pembrolizumab-induced chronic EP. Pembrolizumab was temporarily discontinued, and oral corticosteroids (OCS) at a dose of 30 mg/day (0.5 mg/kg/day) were initiated on an outpatient basis. Soon after starting OCS treatment, the patient’s cough dramatically improved. Subsequently, chest radiography demonstrated a tendency toward improvement in GGO (**Figure 1H**), and blood eosinophil counts and SP-D levels returned to normal within 14 days. Furthermore, FeNO levels rapidly dropped to 59 ppb after starting OCS, along with the improvement of the GGO (**Figure 1I**). Since pembrolizumab had been highly effective for his hypopharyngeal cancer, it was resumed while tapering OCS to a dose of 10 mg/day until day 55. No recurrence of EP has been observed to date, and the therapeutic response of hypopharyngeal cancer has been sustained, with no evidence of tumor progression.

## Discussion

EP is characterized by abnormal eosinophil infiltration due to CD4+ T helper type 2 inflammation in the lungs and prompt response to corticosteroid treatment (6). Although both acute EP and chronic EP share several pathophysiological features, such as marked eosinophilic infiltration in the lung parenchyma and a rapid response to corticosteroid therapy (10), they differ not only in the pathological findings but also clinical presentation and disease course (11). To the best of our knowledge, this is the first report of chronic EP caused by pembrolizumab, one of the ICIs available in clinical practice.

To date, a total of eight cases of EP following ICI administration have been reported, including three cases that were presented in conference papers (12–19) (**Table 2**). The associated ICIs were nivolumab, ipilimumab, or durvalumab, with nivolumab being the most common. The underlying diseases included lung cancer in three cases, and one case each of malignant pleural mesothelioma, renal cell carcinoma, melanoma, esophagogastric adenocarcinoma, and gastric adenocarcinoma. Although the time from ICI initiation to acute EP onset varied, no cases have been reported during the first ICI administration. The diagnosis of acute EP was based solely on BALF analysis in all cases except one, and histopathological examination was not conducted in any of the cases. In the present report, the hallmark pathological findings of chronic EP based on TBLB are presented for the first time, providing valuable insights into ICI-induced EP. Regarding treatment, one previous case was managed solely by discontinuing ICIs, while the others, including the present case, were treated with systemic corticosteroids in addition to ICI discontinuation, all showing significant improvement. However, except for one previous case (13) and the present case, ICI treatment was not resumed. It remains unclear whether the improvement of EP in the present case was mainly due to corticosteroid therapy, ICI discontinuation, or both. Since the number of reported cases is still limited, and treatment responses may vary depending on disease activity, further accumulation of cases is needed to clarify the most effective management approach for ICI-induced EP. However, in patients with symptomatic EP where early resumption of ICI therapy is clinically desirable because of a remarkable response to cancer therapy, corticosteroid treatment may be a reasonable and effective option to achieve prompt symptom relief.

**Table 2:** Reported cases of eosinophilic pneumonia induced by immune checkpoint inhibitors.

Author	Age (year)	Sex	Type of cancer	Type of ICI and number of courses	Maximum eosinophil count (/µL)	CT findings	AEP or CEP	Diagnostic tool	Treatment for EP	Outcome	ICI restart/continuation	Ref. No.
Jodai T (2019)	62	Male	Lung adeno-carcinoma	Nivolumab 3 courses	Not described	Infiltration	AEP	BAL	ICI cessation and OCS 0.5 mg/kg/day	Improvement	Not described	(12)
Hattori Y (2019)	40	Male	Malignant melanoma	Nivolumab 10 courses	853	Infiltration	AEP	Bronchoscopy was not performed	OCS 20 mg/day	Improvement	Continuation	(13)
Hara K (2021)	78	Male	Renal cell carcinoma	Nivolumab and Ipilimumab 4 courses	720	GGO	AEP	BAL	ICI cessation	Improvement	No restart	(14)
Shinada K (2021)	75	Male	Malignant pleural meso-thelioma	Nivolumab 51 courses	853	Infiltration and GGO	AEP	BAL	ICI cessation and mPSL 1000 mg/day for 3 days	Improvement	Not described	(15)
Mouri A (2022)	64	Male	Lung adeno-carcinoma	Durvalumab 21 courses	1,347	Infiltration and GGO	AEP	BAL	ICI cessation and OCS 0.5 mg/kg/day	Improvement	No restart	(16)
Selvan K (2022) *	69	Male	Esophago-gastric adeno-carcinoma	Pembrolizumab (courses are not described)	3,430	Infiltration and GGO	Not described	BAL	mPSL 60 mg/day^†^	Improvement	Not described	(17)
Golden M (2024) *	97	Male	Gastric adeno-carcinoma	Pembrolizumab (courses are not described)	330,000	GGO	AEP	BAL	Prednisone 1mg/kg/day^†^	Improvement	Not described	(18)
J Clark (2024) *	67	Male	Lung invasive mucinous adeno-carcinoma	Pembrolizumab (courses are not described)	3,700	Not described	Not described	BAL	Prednisone 80mg/day^†^	Not described	Not described	(19)

*Published as a conference abstract.

^†^Details regarding ICI cessation were not described.

AEP, acute eosinophilic pneumonia; BAL, bronchoalveolar lavage; CEP, chronic eosinophilic pneumonia; GGO, ground-glass opacity; ICI, immune checkpoint inhibitor; mPSL, methylprednisolone; NA, not applicable;OCS, oral corticosteroids; TBLB, transbronchial lung biopsy; Ref. No., reference number.

Drug-induced ILD (DIILD) associated with ICIs has been reported across all classes of ICIs, with an overall incidence of 3–6%, of which 1–2% were grade 3–4 adverse events as defined by the Common Terminology Criteria for Adverse Events (CTCAE) (20). DIILD encompasses a broad and heterogeneous group of diseases (21). Additionally, the characteristic radiological findings of DIILD show various patterns, including GGO, consolidation, and reticulonodular shadows, making it challenging to distinguish drug-induced EP from other types of drug-induced ILD (22, 23). Therefore, accurate diagnosis of elevated eosinophil counts in BALF via bronchoscopy is crucial not only for determining the need for specific treatment on DIILD, but also deciding whether to continue chemotherapy. However, bronchoscopy may not always be feasible due to the patient’s general condition. In such cases, peripheral blood eosinophil counts and FeNO may serve as useful adjuncts in the diagnosis of EP. Peripheral blood eosinophilia during ICI therapy has been observed with relatively high frequency (approximately 20%) (24). However, some can lead to EP, although there have been extremely limited number of reports of EP or asthma as irAEs associated with ICIs. This discrepancy suggests that eosinophil-associated irAE may be under-recognized or under-reported in clinical practice. Therefore, in cases where peripheral blood eosinophilia is observed during ICI therapy, elevated FeNO levels may serve as a useful surrogate biomarker to support the diagnosis of ICI-associated EP. An increase in FeNO levels occurs when eosinophils infiltrate the airways and lung tissues, where they promote the expression of inducible nitric oxide synthase via interleukin (IL)-13 signaling (25). Previous studies have suggested that FeNO measurement may serve as a substitute for bronchoscopy in diagnosing acute and chronic EP (26), and that FeNO levels can predict patient response to corticosteroid therapy (27). In the present case, both FeNO levels and blood eosinophil counts were markedly elevated at the time of diagnosis, and decreased after the initiation of OCS treatment, which is consistent with the results of previous reports. While these markers may serve as indicators of, and are commonly observed in EP, no biomarkers specific to ICI-induced EP have been established to date. Eosinophilic granule proteins (eosinophil cationic protein, eosinophil derived neurotoxin, eosinophil peroxidase, major basic protein, and galectin-10) have been implicated in EP as other candidate biomarkers, but their predictive value remains uncertain (28). Further research is needed to establish reliable and specific biomarkers for the early detection and risk stratification of ICI-induced EP.

Effective management of ICI-induced EP, an irAE, is crucial for improving prognosis and overall survival. In addition, effective intervention is essential to minimize complications and ensure optimal outcomes. EP typically responds well to systemic corticosteroids, as demonstrated in the present case. Prompt initiation of corticosteroid therapy, along with discontinuation of suspected drugs, is particularly important in cases of DIILD with CTCAE grade 2 or higher, where patients present with respiratory symptoms and abnormal chest radiograph or CT findings. Timely treatment in such cases can reduce the risk of further lung damage and improve patient outcomes. On the other hand, the management of ICI-induced EP raises the issue of rechallenging with ICI. Balancing the need to control EP with the potential benefits of continued cancer treatment remains a challenging aspect of clinical decision-making. A previous analysis of 12 cases of rechallenging ICIs after resolving ICI-induced ILD showed that recurrence occurred in 11% (1/9) of patients with Grade 1 ILD, while the recurrence rate increased to 67% (2/3) in those with Grade 2 ILD (29). In the present case, where the patient had Grade 2 ILD, careful consideration was required before rechallenging with ICIs due to the higher risk of EP recurrence. However, an important point in this case was that pembrolizumab demonstrated high efficacy against hypopharyngeal cancer, which led to the decision to restart the treatment. Moreover, using both FeNO and blood eosinophil levels as non-invasive, adjunctive biomarkers to monitor EP progression may have provided valuable information when considering ICI rechallenge (26). As a result, pembrolizumab was successfully resumed and continued without worsening of EP during OCS treatment while carefully monitoring FeNO and blood eosinophil levels in the present case. This is supported by previous reports that have described cases of ICI-induced asthma, where appropriate asthma management allowed continued ICI treatment (30–32).

The mechanisms by which ICIs induce eosinophilic inflammatory disorders remain unclear. ICIs exert their effects by targeting proteins such as PD-1, PD-L1, or CTLA-4, which enhance T cell activation to mediate robust anticancer responses (2). Among these, pembrolizumab specifically targets PD-1, a receptor on T cells. While blocking the interaction between PD-1 and its ligands [PD-L1 and programmed cell death ligand 2 (PD-L2)], pembrolizumab facilitates the recognition of tumor antigens by tumor-specific T cells, thereby promoting their effector functions to eliminate tumor cells (33). Other PD-1/PD-L1 inhibitors share a similar mechanism of action, leading to the enhancement of T cell–mediated antitumor immunity. However, PD-1 and its ligands (PD-L1 and PD-L2), have also been implicated in allergic diseases (34). Pulmonary dendritic cells express PD-L1 and PD-L2 upon recognition and activation (35). While both ligands regulate airway and lung inflammation, disruption of the interaction between PD-1 and PD-L2 can occasionally promote Th2 inflammation in the lung (36–38). Th2 cells, in turn, produce Th2 cytokines such as IL-4, IL-5, and IL-13, which are critical for activating and recruiting eosinophils to inflammatory sites, potentially causing EP as an irAE. Although pathophysiological mechanisms underlying EP remain poorly understood, this heightened Th2 activity and cytokine release may contribute to eosinophilic activation and inflammation, ultimately leading to EP as an irAE in some patients. Further investigation is needed to elucidate the precise mechanisms underlying ICI-induced EP, through the accumulation of both acute and chronic cases.

In conclusion, not only acute but also chronic EP should be considered as irAEs following ICI therapy. In addition, in cases where ICIs demonstrate significant antitumor efficacy, chronic EP may be manageable with continued ICI administration under careful monitoring of blood eosinophil counts and FeNO levels. Further accumulation of cases is warranted to validate the effectiveness of this approach.

## Data Availability

The original contributions presented in the study are included in the article/supplementary material. Further inquiries can be directed to the corresponding author/s.
